# Predictors of midwifery graduate outcomes in Palestine: a cross-sectional study of curriculum quality, faculty support, and regional disparities

**DOI:** 10.1186/s12912-025-03523-w

**Published:** 2025-07-03

**Authors:** Ibrahim Aqtam, Mustafa Shouli, Saja Aydi, Maysam Morar, Lana Abu Arrah, Amena Abu Rayan

**Affiliations:** Department of Nursing, Ibn Sina College for Health Professions, Nablus University for Vocational and Technical Education, Nablus, Palestine

**Keywords:** Midwifery education, Graduate satisfaction, Employment outcomes, Curriculum quality, Faculty support

## Abstract

**Background:**

Midwifery education critically shapes graduates’ employment status and satisfaction. This study identifies predictors of employment outcomes and satisfaction among Palestinian midwifery graduates, focusing on sociodemographic factors, curriculum quality, clinical training, and faculty support. It also analyzes regional employment disparities and satisfaction variations across graduation cohorts.

**Methods:**

273 graduates of the Midwifery Bachelor’s Program at Ibn Sina College of Health Professions completed a cross-sectional survey. Data collected included sociodemographic information, employment status, and satisfaction with curriculum, clinical training, and faculty support. Instrument validity was confirmed with a Content Validity Index (CVI) of 0.84. A pilot study (*n* = 27) refined the survey. Statistical analyses included Chi-square tests, Pearson’s correlations, and multiple logistic regression, with results presented as odds ratios (OR) and 95% confidence intervals (CI).

**Results:**

Graduates under 25 years had lower employment rates (OR = 0.68, 95% CI: 0.50–0.92, *p* = 0.036), while married graduates were more likely to be employed. Strong faculty mentorship tripled employment odds (OR = 2.75, 95% CI: 1.81–4.18, *p* = 0.002). Curriculum quality moderately correlated with satisfaction (ρ = 0.32, *p* = 0.001). Neonatal training satisfaction was lower (33% excellent, 55% good), with identified gaps in clinical preparedness. Employment disparities across regions were statistically significant (χ² = 10.82, *p* = 0.001), favoring the northern West Bank.

**Conclusion:**

Improvements are needed in neonatal care training and faculty mentorship. Clinical rotations in high-risk settings should expand. Future research must include socioeconomic status and prior healthcare experience as variables. Regional job placement initiatives are necessary to address employment disparities.

**Clinical trial number:**

Not applicable.

## Introduction

Midwifery education is vital for improving maternal and neonatal health outcomes. Midwives reduce morbidity and mortality by providing skilled care across pregnancy, childbirth, and postpartum periods [1]. The WHO stresses that strengthening midwifery education is crucial for achieving Sustainable Development Goals (SDG 3 and SDG 10) [1]. Strong midwifery programs build both technical and interpersonal competencies. Global research highlights the need for curricula that adapt to evolving healthcare needs [2].

In Palestine, midwifery education faces workforce shortages, employment disparities, and role marginalization [3]. Although programs align with international standards, gaps remain [4]. Studies from Jordan and South Africa show that limited neonatal training and gaps in professional development reduce nurses’ clinical competencies and readiness for employment [5, 6]. Palestinian midwifery education must address these weaknesses to meet contemporary demands. There is limited research on regional employment disparities among Palestinian midwifery graduates. Most studies focus on curriculum and training but ignore geographic factors. This study addresses that gap, offering insights into regional workforce inequities.

Midwifery education effectiveness depends on three pillars: curriculum quality, clinical training, and faculty support. Strong curricula combine theory with practical training to build competence [7]. However, Palestinian programs lack specialized neonatal and high-risk obstetric training [4]. Expanding simulation-based learning through partnerships with hospitals and investing in faculty development could strengthen clinical readiness [7]. Clinical training links theory to real-world practice. Graduate satisfaction with placements affects employment outcomes [8]. Yet in Palestine, rural clinical opportunities are limited [9]. Restricted simulation-based training leaves students underprepared for emergencies [10].

Faculty support also shapes education quality. Strong mentorship boosts job readiness and confidence [11]. Palestinian faculty face high teaching loads and limited professional development, reducing their mentorship capacity [12]. Structured mentorship programs could improve graduate preparedness [13]. Regional employment disparities deserve attention. Global studies from sub-Saharan Africa, Southeast Asia, and Latin America demonstrate that midwifery education outcomes are significantly influenced by regional healthcare infrastructure, with rural graduates consistently facing reduced employment opportunities and lower job satisfaction compared to their urban counterparts. Studies in sub-Saharan Africa and Southeast Asia show rural midwifery graduates face fewer jobs and lower satisfaction [14]. This study examines whether similar patterns exist in Palestine and explores solutions for more equitable job distribution. By addressing these issues, this study offers evidence-based recommendations to improve midwifery education, enhance graduate employment, and inform policy reforms.

## Methods

### Study design

This study used a descriptive cross-sectional design to assess experiences of graduates from the Midwifery Bachelor’s Program at Ibn Sina College of Health Professions. Data were collected through a structured online survey measuring curriculum quality, clinical training, faculty support, and employment outcomes. The STROBE checklist guided reporting to ensure methodological rigor [15].

### Participants and setting

Participants were female graduates between 2001 and 2024 who were either currently employed or had prior healthcare experience. Graduates who never worked in healthcare were excluded.

### Sampling strategy

A convenience sampling method was used due to practical constraints. Recruitment included alumni networks, email invitations, and social media outreach to minimize selection bias. Periodic reminders were sent to increase response rates. A total of 273 graduates participated in the study.

### Instrument development and validation

The survey instrument was based on an extensive literature review and WHO midwifery education guidelines [1].

Content validity was assessed using the Content Validity Index (CVI), which yielded a score of 0.84, indicating high item relevance [16].

A pilot study involving 27 participants led to refinement of wording for improved clarity and reliability.

The final questionnaire assessed:

- **Curriculum quality** (theoretical instruction, practical skills, professional competencies).
- **Clinical training** (satisfaction with placements, childbirth and neonatal care exposure, simulation-based learning availability).
- **Faculty support** (mentorship availability, career guidance, instructor engagement).
- **Employment outcomes** (current job status, job relevance to education, perceived employment barriers).

Sociodemographic variables included age, marital status, and graduation year.

Internal consistency reliability was strong, with a Cronbach’s alpha of 0.84 overall, 0.81 for curriculum quality, 0.79 for clinical training, and 0.85 for faculty support.

### Data collection

Data were collected anonymously via a secure online platform (e.g., Qualtrics) between January and March 2024. Participants were informed about the study purpose, confidentiality assurances, voluntary nature of participation, and their right to withdraw at any time. Informed consent was obtained electronically. Survey completion time was estimated at 10–15 min.

### Statistical analysis

Data were analyzed using SPSS Version 26. Descriptive statistics were used to summarize participant demographics and survey responses, including means, standard deviations, frequencies, and percentages. Inferential analyses included Chi-square tests to examine associations between categorical variables (e.g., marital status and employment status), Pearson’s correlation to assess relationships between continuous variables (e.g., curriculum quality and satisfaction), the Kruskal-Wallis test for comparing ordinal data across graduation cohorts, multiple logistic regression to identify predictors of employment outcomes (reporting odds ratios (OR) and 95% confidence intervals (CI)), and multiple linear regression for continuous outcome variables. All statistical tests applied a significance level of *p* < 0.05.

## Results

### Participants’ characteristics

A total of 273 graduates from the Midwifery Bachelor’s Program at Ibn Sina College of Health Professions participated. The mean age of participants was 27.8 years (± 6.4). Nearly half (43%) were under 25 years old, an age group that faced notable employment challenges, as discussed later. Most participants (60%) were married, which appeared to positively influence employment outcomes. Academic performance varied, with the majority (61%) achieving scores between 71 and 80%. As depicted in Table 1: Participants’ Sociodemographic Characteristics (*N* = 273).

**Table 1:** Participants’ sociodemographic characteristics (*N* = 273)

Variable	Category	*N* (%)
Age (years)	< 25	117 (43%)
	25–35	126 (46%)
	> 35	30 (11%)
Mean Age	-	27.8 (± 6.4)
Marital Status	Single	109 (40%)
	Married	164 (60%)
Graduation Year	2001–2019	142 (52%)
	2020	12 (4%)
	2021	8 (3%)
	2022	16 (6%)
	2023	12 (4%)
Grade Rate	65–70%	8 (3%)
	71–80%	167 (61%)
	81–90%	87 (32%)
	> 90%	11 (4%)

### Employment and regional disparities

**Fig. 1:**
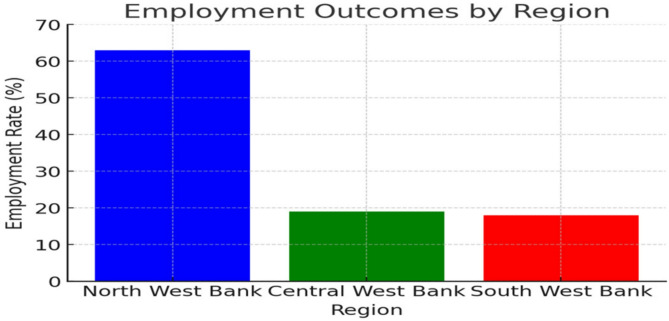
Regional disparities in midwifery graduate employment outcomes (*N* = 273). The figure displays employment rates across three regions of the West Bank, with bars representing the percentage distribution of graduates. Northern West Bank shows 63% employment rate, Central West Bank shows 19%, and Southern West Bank shows 18%, demonstrating clear regional inequities in job opportunities. Satisfaction ratings: Excellent (90–100%), Good (70–89%), Fair (50–69%), Poor (< 50%).

Employment outcomes revealed significant regional disparities (χ² = 10.82, *p* = 0.001); graduates in the northern West Bank had significantly higher employment rates compared to central and southern regions (see Table 2; Fig. 1).

**Table 2:** Employment status and regional distribution (*N* = 273)

Variable	Category	*N* (%)
Employment Status	Employed in Midwifery	164 (60%)
	Employed in Other Health	19 (7%)
	Employed in non-Health	5 (2%)
	Unemployed	85 (31%)
Region of Employment	North West Bank	172 (63%)
	Central West Bank	52 (19%)
	South West Bank	49 (18%)

### Factors influencing educational outcomes

Chi-square tests revealed a significant association between marital status and employment outcomes (χ² = 15.78, *p* = 0.003). A moderate positive correlation was found between curriculum quality and graduate satisfaction (ρ = 0.32, *p* = 0.001). Multiple logistic regression analysis identified key predictors of employment status, showing that graduates under 25 years were less likely to be employed (OR = 0.68, 95% CI: 0.50–0.92, *p* = 0.036), faculty support was the strongest predictor of employment (OR = 2.75, 95% CI: 1.81–4.18, *p* = 0.002), and graduates from the 2022 cohort had significantly better employment outcomes (OR = 1.42, 95% CI: 1.10–1.84, *p* = 0.020) (see Table 3).

**Table 3:** Logistic regression analysis for employment predictors (*N* = 273)

Predictor	OR (95% CI)	*p*-value
Age (< 25 years)	0.68 (0.50–0.92)	0.036
Graduation Year (2022)	1.42 (1.10–1.84)	0.020
Faculty Support	2.75 (1.81–4.18)	0.002

OR = Odds ratio; ci = confidence interval; p-values < 0.05 indicate statistical significance

### Clinical training and graduate satisfaction

Over 54% of graduates rated satisfaction with childbirth training as excellent and 30% as good. However, neonatal training received lower satisfaction ratings, with 33% rating it as excellent, 55% as good, 10% as fair, and 2% as poor. A Kruskal-Wallis test was performed to assess variations in training satisfaction across different cohorts (H = 1.24, *p* = 0.294), revealing no significant differences, suggesting that training quality has remained stable over time (see Table 4).

**Table 4:** Satisfaction with neonatal care training (*N* = 273)

Clinical area	Rating	*N* (%)
Childbirth	Excellent	148 (54%)
	Good	82 (30%)
	Fair	33 (12%)
	Poor	10 (4%)
Neonatology	Excellent	89 (33%)
	Good	151 (55%)
	Fair	27 (10%)
	Poor	6 (2%)

Satisfaction ratings: Excellent = 90–100%, Good = 70–89%, Fair = 50–69%, poor = < 50%

### Satisfaction with childbirth training

**Fig. 2:**
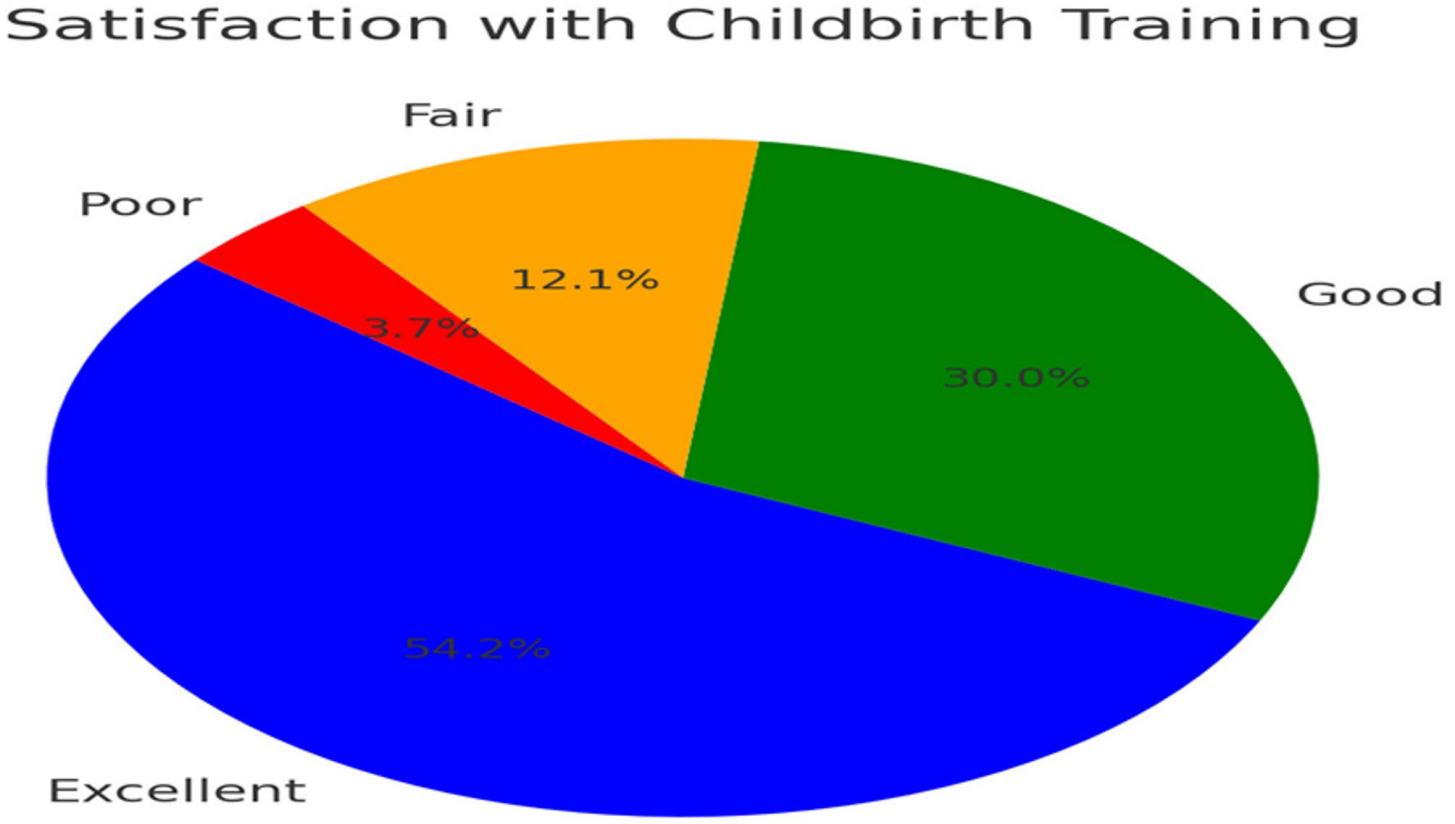
Displays graduate satisfaction with childbirth training using a pie chart format, showing the distribution of satisfaction ratings across the study population

Figure 2 Graduate satisfaction with childbirth training (*N* = 273). The pie chart shows satisfaction ratings distributed as excellent (54%),* good (30%)*,* fair (12%)*,* and poor (4%)*, demonstrating generally positive satisfaction levels with childbirth training components. Satisfaction ratings: Excellent (90–100%),* Good (70–89%)*,* Fair (50–69%)*,* Poor (< 50%).*

### Satisfaction with neonatal training

**Fig. 3:**
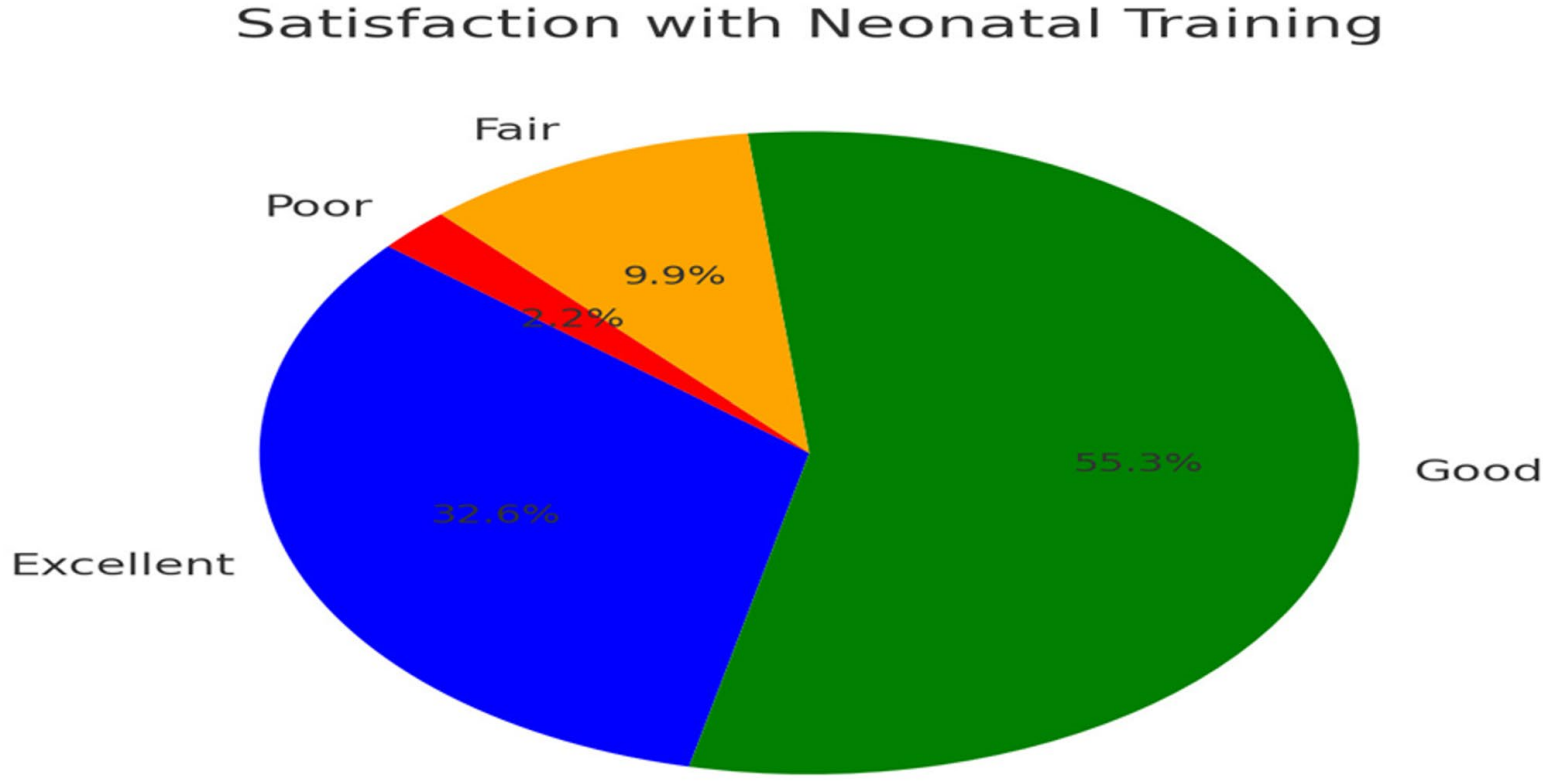
Presents graduate satisfaction with neonatal care training in pie chart format, illustrating the distribution of satisfaction levels across different rating categories

Figure 3 Graduate satisfaction with neonatal care training (*N* = 273). The pie chart displays satisfaction ratings as excellent (33%),* good (55%)*,* fair (10%)*,* and poor (2%). Satisfaction ratings: Excellent (90–100%)*,* Good (70–89%)*,* Fair (50–69%)*,* Poor (< 50%)*.

### Interpretation of non-significant results

While curriculum quality was significantly correlated with satisfaction (ρ = 0.32, *p* = 0.001), clinical training satisfaction did not vary significantly across graduation cohorts (H = 1.24, *p* = 0.294).

## Discussion

This study identified key factors influencing employment outcomes and satisfaction among midwifery graduates in Palestine. Findings confirmed that curriculum quality, clinical training, faculty support, and sociodemographic factors shape employment prospects. This research contributes to the growing body of global literature on midwifery education by providing evidence from a conflict-affected region, complementing studies from established healthcare systems in Europe, North America, and Australia that have consistently demonstrated the importance of structured clinical training and faculty mentorship in graduate outcomes.

### Employment outcomes and sociodemographic factors

Graduates under 25 years faced more employment challenges (OR = 0.68, 95% CI: 0.50–0.92, *p* = 0.036), aligning with global findings that younger healthcare professionals often struggle with initial job placement due to limited experience [8]. Married graduates had higher employment rates, indicating that married graduates had a higher likelihood of employment, aligning with studies on work satisfaction and demographic influences in healthcare professions [17]. Significant regional disparities were evident. Graduates from the northern West Bank secured jobs more frequently (63%) than those from central (19%) or southern (18%) regions (χ² = 10.82, *p* = 0.001). These disparities underscore the urgent need for expanded job opportunities, targeted placement strategies, and healthcare infrastructure improvements in underserved areas. A significant proportion of graduates transitioned into non-midwifery roles, suggesting potential mismatches between training and labor market demands. Similar disparities exist globally, where urban centers attract more graduates, leaving rural areas underserved [14]. To reduce these disparities, policies such as targeted recruitment, financial incentives, and improvements in rural healthcare infrastructure are necessary. Future studies should include socioeconomic status and prior healthcare experience as potential confounding factors.

### Curriculum quality and graduate satisfaction

A strong curriculum is essential for graduate competency and confidence [2]. However, findings revealed critical gaps in neonatal training, where only 33% of graduates rated their experience as excellent. This finding suggests that improvements in curriculum quality enhance overall satisfaction levels. This gap can hinder readiness for high-risk maternal and newborn cases [10]. Simulation-based training improves clinical confidence and performance. Studies show that high-fidelity simulation significantly enhances preparedness for emergencies [7]. Palestinian programs should integrate advanced simulation in neonatal resuscitation and expand clinical rotations in neonatal intensive care units (NICUs).

### Faculty support and employment readiness

Structured faculty mentorship was found to be the strongest predictor of employment (OR = 2.75, 95% CI: 1.81–4.18, *p* = 0.002), confirming that structured academic mentorship improves job readiness and professional development [11]. However, Palestinian midwifery faculty face heavy workloads and limited professional development opportunities [12]. Improving faculty capacity through formal mentorship programs, reduced teaching loads, and investment in faculty training workshops can significantly enhance student support and graduate employability.

#### Interpretation of non-significant results

However, this non-significant result does not imply equivalence between cohorts; it may reflect uniformity in training protocols or methodological limitations (e.g., survey sensitivity to detect nuanced differences). Qualitative investigations are needed to explore graduates’ specific experiences and identify latent gaps in clinical training.

### Strengths and limitations

#### Strengths

This study is the first to systematically examine midwifery graduate employment outcomes in Palestine, featuring a large, representative sample (*N* = 273) covering various cohorts and regions. It also demonstrates high internal consistency of measurement tools, with a Cronbach’s alpha of 0.84.

#### Limitations

The study has several limitations: convenience sampling may introduce bias despite broad recruitment strategies; self-reported data can suffer from recall bias; the cross-sectional design limits causal interpretations; and the study did not measure socioeconomic status or prior healthcare experience, which could influence employment outcomes.

#### Recommendations

Future studies should include socioeconomic status and prior healthcare experience as potential confounding factors.

Palestinian programs should integrate advanced simulation in neonatal resuscitation and expand clinical rotations in neonatal intensive care units (NICUs).

Improving faculty capacity through formal mentorship programs, reduced teaching loads, and investment in faculty training workshops can significantly enhance student support and graduate employability.

## Conclusion

This study identified key factors shaping the employment outcomes and satisfaction of midwifery graduates from Ibn Sina College of Health Professions, with curriculum quality, clinical training, and faculty support emerging as critical components of graduate success. Faculty mentorship was found to be the strongest predictor of employment, highlighting the importance of structured, career-oriented guidance within midwifery education. Additionally, regional employment disparities were significant, with northern West Bank graduates more likely to secure jobs than their central and southern counterparts. To address these challenges, targeted strategies are necessary: curriculum reforms should strengthen neonatal care and high-risk obstetric training, integrating simulation-based education to improve clinical competence; mentorship programs should be formalized, pairing students with experienced midwives for sustained academic and career support; and regional job placement initiatives and financial incentives must be implemented to encourage graduates to work in underserved areas. Future research should incorporate factors such as socioeconomic status, prior healthcare experience, and geographic mobility as additional predictors of employment, while longitudinal tracking of graduates would provide deeper insights into employment trajectories and inform evidence-based educational reforms. By addressing these gaps, Palestinian midwifery programs can enhance graduate employability, ensure equitable workforce distribution, and contribute to improved maternal and neonatal health outcomes.

## Data Availability

The datasets generated and analyzed during this study are not publicly available to protect participant confidentiality. De-identified data may be made available from the corresponding author upon reasonable request, subject to approval by the Ibn Sina College of Health Professions Institutional Review Board (IRB).
